# COVID-19 Vaccine Acceptance among College Students: A Theory-Based Analysis

**DOI:** 10.3390/ijerph18094617

**Published:** 2021-04-27

**Authors:** Manoj Sharma, Robert E. Davis, Amanda H. Wilkerson

**Affiliations:** 1 Department of Environmental and Occupational Health, University of Nevada, Las Vegas, NV 89119, USA; manoj.sharma@unlv.edu; 2 Substance Use and Mental Health Laboratory, Department of Health, Human Performance and Recreation, University of Arkansas, Fayetteville, AR 72701, USA; red007@uark.edu; 3 Department of Human Environmental Sciences, The University of Alabama, Tuscaloosa, AL 35487, USA

**Keywords:** multi-theory model, MTM, vaccine, pandemic, vaccine uptake, vaccine acceptability, young adults, college students, COVID-19

## Abstract

The severity and pervasiveness of the COVID-19 pandemic have necessitated the emergency use of COVID-19 vaccines. Three vaccines have been approved in the United States (USA). However, there is still some hesitancy in COVID-19 vaccine acceptability among some subgroups, including college students. While research is limited on vaccine acceptability behavior among college students, preliminary data suggests hesitancy as being high. This study aimed to explain the correlates of COVID-19 vaccine acceptance among college students who reported hesitancy toward the COVID-19 vaccine and those who did not using the initiation component of the multi-theory model (MTM) of health behavior change. Using a cross-sectional study design, data were collected from a Southern USA University (*n* = 282) utilizing a valid and reliable 27-item questionnaire in February and March 2021. Almost half (47.5%) of participants reported hesitancy to receive the COVID-19 vaccine. The three constructs of MTM’s initiation model, behavioral confidence (*b* = 0.089, *p* < 0.001), participatory dialogue (*b* = 0.056, *p* < 0.001), and changes in the physical environment (*b* = 0.066, *p* = 0.001) were significantly associated with COVID-19 vaccine acceptance among those who were not hesitant to take the vaccine and accounted for 54.8% of the variance. Among those who were hesitant to take the COVID-19 vaccine, the MTM construct of behavioral confidence (*b* = 0.022, *p* < 0.001) was significant along with Republican Party political affiliation (*b* = −0.464, *p* = 0.004), which was negatively associated with vaccine acceptance. The model accounted for 60.6% of the variance in intention to take the COVID-19 vaccine. This study provides evidence for the utility of MTM as a timely intervention to design messages for college students to enhance COVID-19 vaccine acceptability.

## 1. Introduction

The novel coronavirus (COVID-19) pandemic caused by the widespread impacts of the SARS-Cov-2 virus has drastically impacted health, economics, and individual lives worldwide [1,2]. Notably, the effects of the COVID-19 pandemic in the United States (US) have been drastic. As of 12 March 2021, the US has documented over 500,000 deaths and has a higher mortality rate (157.8 per 100,000) and morbidity rate (87.4 per 1000) compared to most other developed countries worldwide [3]. The mortality rate and severe impact of COVID-19 in the US underscores the importance of vaccination against COVID-19. Currently, there are three vaccines approved for Emergency Use Authorization by the US Food and Drug Administration (FDA): Pfizer–BioNTech (BNT161b2), Moderna (mRNA-1273), and Janssen (Ad26.COV2.S; FDA, 2021). In clinical trials, all three vaccines have demonstrated high efficacy in preventing lab-diagnosed COVID-19 infection (66% for Janssen; 95% and 94.1% for Pfizer–BioNTech and Moderna, respectively [4,5,6]. As of 12 March 2021, 10.2% of US adults are fully vaccinated and 19.3% have received one dose of the COVID-19 vaccine [7]. The US government has intentions for the entire adult population in the US to have an opportunity to receive a vaccine as early as May 2021 [8].

Data collected by the Pew Research Center in February 2021 indicate that approximately 69% of US adults plan to receive the COVID-19 vaccine, an increase from 60% in November 2021 [9]. However, public health officials remain concerned with the public’s perception of the vaccine and the acceptability and intention to receive the vaccination, especially in certain demographic sub-groups [10,11]. Recent research indicates that certain population subgroups, including people with lower income levels, conservative political affiliations, certain religious beliefs, women, people with Black and Hispanic racial identity, and young adults [9,10,12]. Multiple factors contribute to vaccine hesitancy among US adults, including fear of potential side effects, previous experiences with healthcare providers, social acceptance of peers, negative perceptions of vaccine manufacturers (i.e., pharmaceutical companies), belief in conspiracy theories, concern about the rapid development of the vaccines, and lack of trust in government policymakers [9,13].

An important group of young adults to target for COVID-19 vaccination are college students. College students are vulnerable to SARS-Cov-2 infection due to a multitude of factors: communal residency in on-campus and off-campus housing, the reopening of college campuses and activities, and the necessity to travel between their home and campus. Furthermore, college campuses have been identified as at risk to develop cases of COVID-19 and have the potential to become “superspreaders” with likely impacts on neighboring communities [14]. Research among college students in Italy regarding COVID-19 vaccine intention shows that certain student sub-groups may have greater vaccination intention, including medical students, students with previous uptake of flu vaccination, students with a higher level of concern about COVID-19, and students with high vulnerability to COVID-19 [15]. Furthermore, recent low flu vaccination trends among younger US adults (ages 18 to 49) compared to other age groups warrants the need to investigate US college students’ perceptions and intentions to accept a COVID-19 vaccine once it is available [16]. However, minimal research has assessed vaccine intentions among US college students. To create effective strategies to increase COVID-19 vaccination, it is imperative to understand the factors that contribute to COVID-19 vaccine intention and behavior, especially in demographic subgroups with potential hesitancy or low uptake of COVID-19 vaccines, such as some groups of college students.

The present study was based on a theoretical frame of the multi-theory model (MTM) for health behavior change to explore COVID-19 vaccination intentions among college students in the US. The MTM is unique in the theory’s ability to explain not only the intention but also the sustenance of behavior change [17,18]. The theory’s constructs are separated by the initiation (i.e., one-time engagement in a health behavior) and sustenance (i.e., long-term engagement in a health behavior) outcomes for behavior change. The theory uses three constructs to explain initiation and three additional constructs to explain sustenance of a health behavior. For this study, only the initiation model and associated constructs were assessed as vaccine uptake is a one-time behavior (i.e., administered in one or two doses) and not a long-term behavior change. For initiation, the MTM suggests that three constructs: participatory dialogue (i.e., whereby the advantages of a behavior change are seen as being more than disadvantages), behavioral confidence (i.e., whereby the person becomes sure in performing any given behavior), and changes in the physical environment (i.e., whereby the person has necessary resources for performing a given behavior) will lead to initiation in a behavior change [17,18]. The MTM is unique in that the constructs can be applied across different health behaviors, and the theory has been used to explain a diverse range of health behaviors [19,20,21,22,23,24]. The MTM has advantages over previous health behavior theoretical models, which have yielded mixed findings about behavior change, are shown to lack substantive predictive power, and typically are not situated around long-term behavior change [17].

The purpose of this study was to explain COVID-19 vaccine initiation among college students who reported hesitancy towards the COVID-19 vaccine and those who did not, using a theory-based approach with the MTM. The findings from this study yield important information to inform the development of health promotion programming, messaging, and interventions to increase COVID-19 vaccine uptake among college students. The exploration of factors related to initiation for the two groups (i.e., college students who are hesitant and those who are not) may assist health promotion researchers and practitioners to created targeted and tailored messaging, especially for students reporting hesitancy towards the COVID-19 vaccine.

## 2. Materials and Methods

### 2.1. Study Design

The study used a quantitative cross-sectional and survey-based research methodology. The questionnaire was administered to a sample of University students at a Southern US University. The design was chosen because of its ease of administration, cost, and rapid data collection, which was needed due to the urgency of this research and the anticipated vaccine availability for all US adults [8,25]. The independent variables in the study were the constructs of MTM from the initiation model, while the dependent variable was the intention for taking a COVID-19 vaccination. Study participants were divided into two groups: those who expressed hesitancy toward the COVID-19 vaccine and those who did not have any hesitancy. Inclusion criteria for participation in the study were: (1) current enrollment at the University; (2) age 18 years or older; (3) having access to the Internet; (4) providing informed consent; and (5) ability to communicate in English.

### 2.2. Survey Instrument

The survey instrument was developed by the first author (see Supplementary Materials). The instrument consisted of one item about vaccine hesitancy (i.e., If the COVID-19 vaccine was offered to you today, do you have any hesitancy in taking it?), 13 demographic items (e.g., age, gender, ethnicity, race, etc.) three items pertaining to the advantages construct (viz. protection against COVID-19, protection of family, and resuming activities), three items pertaining to the disadvantages construct (viz. about safety, lack of long-term studies, becoming ineffective due to mutation), three items pertaining to the behavioral confidence construct (e.g., surety of taking it, taking it despite side effects, despite worries that long-term studies have not been done), three items pertaining to changes in the physical environment construct (e.g., accessibility, affordability, convenience), and one item about the intention to take COVID-19 vaccine. It was then validated for face and content validity through a panel of six experts in two rounds. All experts had doctorates in public health and were an expert in one or more of the following: (1) instrumentation; (2) COVID-19; (3) target population (e.g., college students); and (4) MTM. As a result of the validation, a 27-item instrument with a Flesch–Kincaid Grade Level of 5.9 and Flesch Reading Ease score of 63.5 (overall) was finalized. The instrument was informally pilot tested with a group of college students for readability and comprehension and was found to be easily understood. The instrument was administered via Qualtrics, an online survey tool.

The study was approved under the exempt category by the Institutional Review Board at the University of Arkansas (IRB Protocol #2102312641), dated 17 February 2021. All students were recruited via a post in the University’s daily e-news bulletin. The landing page of the electronic survey instrument provided informed consent and study information. The landing page included specific information about the study, the principal investigator’s contact information, the purpose of the study, the voluntary nature of participation, IRB contact information, and information about the rights of the participants to withdraw at any point in time. Personal identifiers (i.e., IP addresses, names, emails) were not collected during survey administration. Participants were instructed that by clicking the next button, they were consenting to all information provided and participation in the study.

### 2.3. Sample Size and Data Collection

An a priori sample size was calculated by G*Power, Version 3.1 [26] using the following parameters: alpha at 0.05, power at 0.80, an estimated medium effect size of 0.10 [27], and three predictors, which yielded a sample size of 114. In case additional covariates were found to be significant, the sample size was inflated to around 135 for each of the two groups (e.g., hesitant and non-hesitant). It was assumed that the two groups would be equal. Data were collected between February and March 2021 using Qualtrics online survey software. Students were recruited through advertisements in the University’s daily e-news bulletin, which is sent weekly to all enrolled students with a valid university email. The advertisements were posted on Tuesdays and Thursdays for three consecutive weeks. The Qualtrics questionnaire termination options were set to mitigate “Ballot Box Stuffing.” This prevented the respondents’ ability to complete the survey twice from the same IP address. As an incentive, students were informed that, by participating, they would be eligible to enter a drawing to receive one of five gift cards valued at $20.00 USD The final item of the Qualtrics instrument served as a link to a separate survey instrument where participants were able to enter their preferred email address for contact purposes for the gift card drawing. This process ensured that prior data collected could not be linked to individual email addresses, ensuring participant anonymity.

### 2.4. Statistical Analyses

The data from Qualtrics were downloaded as a Microsoft Excel file and then imported into SPSS (Version 26.0, IBM, Armonk, NY, USA) for data analyses. Construct validity of the four MTM subscales (i.e., advantages, disadvantages, changes in the physical environment, and behavioral confidence) was determined by confirmatory factor analysis with maximum likelihood estimation. Using this method, each subscale yielded a single-factor solution, with all factor loadings over 0.32, which is double the critical value for a correlation coefficient at α = 0.01 for a two-tailed test, and all Eigenvalues were greater than 1.0 [28]. For establishing internal consistency reliability Cronbach’s alphas were computed for each subscale and were found to be over 0.70, which is deemed acceptable [25]. Descriptive statistics for qualitative variables (i.e., frequencies and percentages) and for continuous variables (i.e., means and standard deviations) were computed and reported. The independent variables were the constructs of MTM, and the dependent variable was the intention to take the COVID-19 vaccine (e.g., initiation in the MTM).

Demographic variables exhibiting a significant bivariate relationship to the outcome variable served as covariates in the multivariate models. The covariates of religion and political affiliation were constructed based on graphing the response distributions of initiation within each level of religion and political affiliation, followed by mean testing with post hoc tests to confirm differences. Republican Party political affiliation was significantly lower on the intention scale than other affiliations. Similarly, intention means for Christianity and Islam were nearly identical and significantly differed from each of the other religions. No other significant differences were observed between religious affiliations or between the three other political affiliations. A zero-order correlation matrix of study variables was prepared to identify relationships between theoretical constructs in both groups. Hierarchical multiple regression modeling was employed for the hesitant group as well as the non-hesitant group, yielding two separate models. The significance level was set a priori at α = 0.05 for all analyses.

## 3. Results

In total, 416 students accessed the invitation to participate in the current study. Among them, 126 did not meet the specific inclusionary criterion of having not received a single dose of the COVID-19 vaccine. Thus, the final sample included 282 participants (Table 1). The mean age of participants was 25.00 ± 7.90 years. The majority identified as female (62.8%) and White (56.4%). The largest representation by academic classification was observed among graduate students (28.4%) and undergraduate seniors (26.2%). More than half of respondents held a grade point average of at least 3.00 out of 4.00, with 48.2% of students working at least part-time and 70% of students residing in off-campus housing. Participants identified their political affiliation as Republican, Democrat, Independent, and other, at 17.0%, 37.2%, 26.2%, and 14.5%, respectively. Further, religious affiliation was predominantly Christian (60.3%). The vast majority of participants possessed health insurance (91.1%). Importantly, 47.5% of individuals reported that they were hesitant to receive the vaccine, and only 50% reported that they had been encouraged by a healthcare provider to receive the vaccine when it became available to them.

Table 2 documents the descriptive characteristics of MTM variables and provides significance testing between individuals reporting hesitancy and those reporting no hesitancy to receive a COVID-19 vaccine. Cronbach’s alpha for the variables was at or above 0.75 for all variables among both groups, indicating adequate internal consistency among measures of the theoretical constructs. Except for the participatory dialogue construct, which was significantly lower among hesitant individuals, mean values for theory measures were significantly higher among individuals exhibiting no hesitance in vaccine acceptance.

The bivariate associations between MTM constructs were assessed among hesitant and non-hesitant participants (Table 3). Consistent with theory logic, initiation was significantly related to participatory dialogue: advantages−disadvantages (*r* = 0.338, *p* < 0.001), behavioral confidence (*r* = 0.764, *p* < 0.001), and changes in the physical environment (*r* = 0.349, *p* < 0.001) for hesitant individuals. Similarly, among non-hesitant individuals participatory dialogue: advantages−disadvantages (*r* = 0.534, *p* < 0.001), behavioral confidence (*r* = 0.670, *p* < 0.001), and changes in the physical environment (*r* = 0.606, *p* < 0.001) significantly associated with the initiation construct. Apparent are the increased magnitude of associations between initiation of vaccination and the constructs participatory dialogue and changes in the physical environment between non-hesitant individuals and their hesitant counterparts.

To further examine the MTM’s ability to explain vaccine acceptance, multiple regression modeling was conducted (Table 4). In addition to theory-based predictors, individual characteristics exhibiting a significant bivariate relationship with the outcome variable (i.e., initiation of vaccination), within hesitant and non-hesitant groups, were included as covariates in the constructed multivariate models. Among hesitant participants, multiple regression modeling accounted for approximately 60% of the variability in initiation of vaccination (adjusted *R*^2^ = 0.606, *F*_(5,121)_ = 39.68, *p* < 0.001). Following covariate adjustment, behavioral confidence (*b* = 0.222, *p* < 0.001) remained significantly associated with initiation. In addition to theory-based predictors, one individual characteristic emerged as a significant predictor of initiation, following adjustment. Specifically, a Republican Party political affiliation equated to a 0.464 reduction in initiation score (*b* = −0.464, *p* = 0.004) when compared to those endorsing non-republican political affiliations (i.e., Democrat, Independent, or other).

Among non-hesitant individuals, a significant regression model also emerged explaining 54.8% of the variance in initiation. Herein, participatory dialogue: advantages−disadvantages (*b* = 0.056, *p* < 0.001), behavioral confidence (*b* = 0.089, *p* < 0.001), and changes in the physical environment (*b* = 0.066, *p* = 0.001) all remained significant predictors of initiation following covariate adjustment. Among non-hesitant individuals, gender exhibited a significant bivariate relationship with initiation. The significance of this relationship was nullified in the multivariate model.

## 4. Discussion

This study aimed to explain the correlates for COVID-19 vaccine acceptance among college students who reported hesitancy towards the COVID-19 vaccine and those who did not using the initiation component of the MTM. As expected, the three constructs of the MTM’s initiation model, namely participatory dialogue, behavioral confidence, and changes in the physical environment, were significantly associated with COVID-19 vaccine initiation among those who were not hesitant to take the vaccine and accounted for 54.8% of the variance. This finding confirmed the salience of these constructs in COVID-19 vaccine initiation. Furthermore, the β that was the largest in the model was that of behavioral confidence (0.373), underscoring the importance of this construct. Among those who were hesitant to take the COVID-19 vaccine, the MTM construct of behavioral confidence was once again significant along with Republican Party political affiliation, which was negatively associated with vaccine acceptance. The model accounted for a considerable proportion of the variance in the intention to take the COVID-19 vaccine which is fairly high in behavioral and social science research [25].

In our sample, 47.5% of the individuals reported hesitancy to receive the COVID-19 vaccine. This finding is slightly lower than the finding of Laitkin et al. [10] who reported 60.6% of young adults ages 18–29 years either expressing they were not sure or did not intend to receive the COVID-19 vaccine. This may indicate a trend toward acceptance of the COVID-19 vaccine as more people continue to be vaccinated, but the need to convince a large proportion of college students remains an important public health issue, as there was still a considerable portion of our sample that expressed hesitancy towards the vaccine.

Examining each construct of the MTM, based on this study, provides greater insight into the perceptions of college students and how to alter the essential constructs through public health education and policy efforts. The construct of participatory dialogue, which is based on value-expectancy theories [18], was found to be significant for those who were not hesitant but not significant for those who were hesitant. Furthermore, a comparison of mean scores between the two groups yielded a significant difference for the hesitant group when compared to those who were non-hesitant. The low levels of belief in the advantages of the COVID-19 vaccine in the hesitant group point to the need for health promotion programming to convince this group of the advantages of vaccination not only for their benefit but also the greater benefits to society [14].

The construct of behavioral confidence, or an individual’s sureness to properly follow through with receiving the COVID-19 vaccine despite obstacles, was significant for both hesitant and non-hesitant groups and accounted for substantial contributions in both models. For the hesitant group, for every one-unit increase in behavioral confidence, the intention to accept the COVID-19 vaccine increased by 0.22 units on a scale of 0 to 4 units. It is worth noting that the mean for this group was 1.55 ± 1.21 units, so such contribution is quite significant. For the non-hesitant group, for every unit increase in behavioral confidence, the intention to accept vaccine increased by 0.373 units on a scale of 0 to 4 units. Behavioral confidence has been found to be an important construct in many previous studies conducted using MTM with other health behaviors in college students [21,22,29] and other populations [19,24]. Influencing behavioral confidence should be an integral part of all health promotion efforts planned with college students to enhance COVID-19 vaccine uptake. Public health researchers and practitioners should consider incorporating strategies to address behavioral confidence to enhance COVID-19 vaccine uptake, such as including information related to common barriers or myths surrounding the COVID-19 vaccine in messaging encouraging vaccine uptake in this population.

The construct of changes in the physical environment was found to be significant for those who were not hesitant but not significant for those who were hesitant. Furthermore, a comparison of the two groups yielded a significant difference among the mean scores for the hesitant group as compared to the mean scores for the non-hesitant group. With the recent commitment by the Biden administration to making vaccination available for the entire adult population in the US by May 2021, this construct would lose its importance to some extent [8]. The only factors that would remain would be in terms of convenience, transportation, place of administration, and barriers to access associated with receiving the vaccine. Once vaccines are available to all US adults, policymakers will have to ensure that these barriers are reduced in order to ensure most college students have an opportunity to receive the vaccine.

The study also found that a Republican Party political affiliation was associated with increased hesitancy to receive the vaccine. This finding has also been supported in the literature, where other researchers have shown affiliation with the Republican Party, or conservative political beliefs, to be associated with decreased likelihood to receive the COVID-19 vaccine and hesitancy towards the vaccine [9,10]. The association between political affiliation and COVID-19 vaccination hesitancy may be due to the current politicization as well as polarization of COVID-19 and the vaccine in the US. Thus, this relationship may be unique given the current political climate [10]. Future research should explore vaccine hesitancy and uptake to determine if this relationship persists. Due to the associations between certain political affiliations and vaccine hesitancy, it would behoove the leaders in the Republican Party to serve as role models and also actively advocate for receiving the COVID-19 vaccine, which would encourage and motivate certain college students to receive the COVID-19 vaccine.

In terms of other demographic variables, it is worth noting that only 50% of the college students in our sample reported being encouraged by their healthcare provider to receive the COVID-19 vaccine. This finding points to the need for each healthcare provider to emphasize to their patients to receive the COVID-19 vaccine. It has been shown that people tend to listen more to the advice given by healthcare providers [30,31]. Other demographic variables such as living arrangement, ethnicity, academic classification, and employment status were found not to contribute to the predictive models in this study but may be important to investigate in future research in the college student population.

The findings from this study are important for public health professionals, health education specialists, policymakers, college educators, University wellness centers, healthcare providers, campus-based student organizations, and college students. Public health professionals, health education specialists, and healthcare providers should consider designing educational programs and messaging that promotes behavioral confidence among college students to receive the COVID-19 vaccine. Healthcare providers can play a strong role in this process. Such messaging could also utilize role models for college students, including peers and people that college students look up to. It would behoove universities to organize the publicizing of the vaccination behaviors of prominent University sub-community leadership. In addition to standard University administrative figures, leaders among the Greek communities, athletics, and other registered student organizations (RSO) can be effective means to influence behavior among the broader student body. These messages should be appealing to college students and must be brief and clear. Social media sites can also be utilized to deliver these messages in addition to official university outlets, such as daily news bulletins, student organizations, and campus wellness centers. There is also a need to correct any misinformation or fake news that may be circulating around the COVID-19 vaccine for college students.

Besides educational interventions, there is also a need for policymakers, both at the university level and at the national level, to promote COVID-19 vaccine acceptance among this high-risk and reluctant group of young adults. At the university level, policies should be mandated to inform and educate the students while, at the national level, barriers associated with vaccine uptake should be counteracted along with a national campaign to promote COVID-19 vaccine acceptance in this group.

To our knowledge, this is the first theory-based study to enhance understanding of COVID-19 vaccine uptake behavior among college students. This study utilizes a contemporary fourth-generation model, MTM, and helps improve theoretical operationalization for other researchers. The valid and reliable instrument developed in this study (see Supplementary Material) can be used in designing and testing public health education interventions. The study also had some limitations. For example, data were collected from a single institution which limits the generalizability of our findings. Additionally, the sample contained predominantly female, upperclassmen, and graduate students. This further limits the generalizability of the study findings to other groups of college students. Since the study was completed before the COVID-19 vaccine was available to most college students, we could only assess their intention of taking the vaccine and not the actual behavior. Regarding the temporality of the measurement scale used in the study, we could not conduct test–retest reliability on the scale because of the need for rapid dissemination of our findings.

## 5. Conclusions

The present study examined the utility of MTM in explaining COVID-19 vaccine acceptability among college students. The MTM offers a simple and pragmatic context for designing educational interventions for college students that can promote COVID-19 vaccine acceptance. This is the right time in the COVID-19 pandemic era to design and disseminate evidence-based interventions to promote COVID-19 vaccine acceptability for college students, as most college students will soon be eligible to receive a COVID-19 vaccine. Based on this study, it can be concluded that MTM can be an effective tool to develop public health education programming and messaging to encourage vaccine uptake among college students with varying levels of vaccine hesitancy.

## Figures and Tables

**Table 1:** Descriptive characteristics of the study sample (*n* = 282)**.**

Characteristic	M (SD)	*n* (%)
Age	25.00 (7.90)	
Gender		
Female		177 (62.8)
Male		95 (33.7)
Ethnicity		
White/Caucasian		159 (56.4)
Black/African American		15 (5.3)
Asian		23 (8.2)
American Indian/Alaskan Native		21 (7.4)
Latino/Hispanic		37 (13.1)
Other		5 (1.8)
Academic classification		
Freshman		33 (11.7)
Sophomore		47 (16.7)
Junior		39 (13.8)
Senior		74 (26.2)
Graduate student		80 (28.4)
Grade point average		
<1.99		6 (2.1)
2.00–2.49		15 (5.3)
2.50–2.99		56 (19.9)
3.00–3.49		71 (25.2)
3.50–4.00		120 (42.6)
Employment		
No		130 (46.1)
Yes (hours per week)	17.33 (10.71)	136 (48.2)
Living arrangement		
Off-campus		194 (68.8)
On-campus		72 (25.5)
Political affiliation		
Republican		48 (17.0)
Democrat		105 (37.2)
Independent		74 (26.2)
Other		41 (14.5)
Religion		
Christianity		170 (60.3)
Islam		24 (8.5)
Buddhism		12 (4.3)
Judaism		5 (1.8)
Hinduism		33 (11.7)
Other		21 (7.4)
Health insurance		
No		11 (3.9)
Yes		257 (91.1)
Hesitancy to receive COVID-19 vaccination		
No		145 (51.4)
Yes		134 (47.5)
Encouraged by HCP to receive COVID-19 vaccination		
No		130 (46.1)
Yes		141 (50.0)

Percentages may not total 100% due to missing data in the form of participant omission.

**Table 2:** Descriptive statistics for study variables with the test of group means between vaccine-hesitant and non-hesitant individuals.

Variable	Vaccine-Hesitant Individuals (*n* = 134)				Vaccine Non-Hesitant Individuals (*n* = 145)				
	Possible Range	Observed Range	Mean (SD)	Cronbach’s Alpha	Possible Range	Observed Range	Mean (SD)	Cronbach’s Alpha	*p*-Value
Initiation	0–4	0–4	1.55 (1.21)	-	0–4	1–4	3.56 (0.74)	-	<0.001 *
Participatory dialogue: advantages	0–12	0–12	6.95 (3.43)	0.93	0–12	4–12	9.91 (2.01)	0.77	<0.001 *
Participatory dialogue: disadvantages	0–12	0 – 12	7.63 (2.99)	0.88	0–12	0–12	4.62 (2.38)	0.75	<0.001
Participatory dialogue: advantages–disadvantages	−12–+12	−12–+8	−0.68 (3.81)	-	−12–+12	−3–+12	5.30 (3.49)	-	<0.001
Behavioral Confidence	0–12	0–12	3.92 (3.41)	0.90	0–12	0–12	9.33 (3.13)	0.91	<0.001
Changes in the physical environment	0–12	0–12	5.19 (3.66)	0.92	0–12	0–12	8.19 (3.12)	0.78	<0.001 *

Estimates attained for significance testing are based on Student’s *t*-tests. * Indicates Welch’s *t*-test.

**Table 3:** Zero-order correlation matrix of study variables.

**Vaccine Hesitant Individuals (*n* = 134)**					
**Construct**		**1**	**2**	**3**	**4**
1.	Initiation	-	0.338 *	0.764 *	0.349 *
2.	Participatory dialogue advantages–disadvantages		-	0.291 *	0.153
3.	Behavioral confidence			-	0.421 *
4.	Changes in the physical environment				-
**Vaccine Non-Hesitant Individuals (*n* = 145)**					
**Construct**		**1**	**2**	**3**	**4**
1.	Initiation	-	0.534 *	0.670 *	0.606 *
2.	Participatory dialogue advantages−disadvantages		-	0.459 *	0.366 *
3.	Behavioral confidence			-	0.612 *
4.	Changes in the physical environment				-

* *p* < 0.001.

**Table 4:** Multiple regression models for Initiation of vaccination among hesitant and non-hesitant individuals.

**Hesitant Individuals**	***b***	**S.E.**	**β**	***p***	**LBCI**	**UBCI**
Encouraged by HCP *	0.001	0.135	0.00	0.993	−0.266	0.268
Republican (reference: non-republican)	−0.464	0.158	−0.175	0.004	−0.776	−0.151
Participatory dialogue: advantages–disadvantages	0.031	0.018	0.103	0.088	−0.005	0.066
Behavioral confidence	0.222	0.022	0.669	<0.001	0.178	0.265
Changes in the physical environment	0.018	0.019	0.060	0.341	−0.020	0.056
Model statistics: Adjusted *R*^2^ = 0.606, *F*_(5,121)_ = 39.68, *p* < 0.001						
**Non-Hesitant Individuals**	***b***	**S.E.**	**β**	***p***	**LBCI**	**UBCI**
Gender	0.020	0.093	0.014	0.826	−0.164	0.204
Participatory dialogue: advantages−disadvantages	0.056	0.014	0.262	<0.001	0.027	0.084
Behavioral confidence	0.089	0.019	0.373	<0.001	0.052	0.127
Changes in the physical environment	0.066	0.018	0.272	0.001	0.029	0.102
Model Statistics: Adjusted *R*^2^ = 0.548, *F*_(4,126)_ = 40.37, *p* < 0.001						

* HCP = health care professional. S.E. = standard error of the estimate; LBCI = lower bound of the 95% confidence interval; UBCI = upper bound of the 95% confidence interval.

## Data Availability

The data presented in this study are available on request from the corresponding author. The data are not publicly available due to participant confidentiality associated with the ethical approval received for this study.

## Supplementary Materials

The following are available online at https://www.mdpi.com/article/10.3390/ijerph18094617/s1, Supplementary File S1: Measuring COVID-19 Vaccine Acceptance Behavior.
