# Supplementary material for: COVID-19 Vaccine Acceptance among College Students: A Theory-Based Analysis
Source: Int J Environ Res Public Health. 2021 Apr 27;18(9):4617. doi: 10.3390/ijerph18094617 (PMC8123652; doi:10.3390/ijerph18094617)
Supplement: Supplementary file 1 [file ijerph-18-04617-s001.zip › ijerph-1166595-supplementary.pdf]

SUPPLEMENTARY MATERIAL: MEASURING COVID-19 VACCINE ACCEPTANCE BEHAVIOR

Directions: This survey is voluntary, which means you may choose not to complete it or not to answer individual questions. There is no direct benefit of this survey to you but your responses will help in developing effective COVID-19 vaccine promotion programs. All personal data from this survey will be kept confidential. If you agree, please choose the response that correctly describes your position. Thank you for your help!

1. If the COVID-19 vaccine was offered to you today, do you have any hesitancy in taking it? ☐ Yes ☐ No

…………………………………………………………………………………………………

2. How old are you today? _______ years

…………………………………………………………………………………………………

3. What is your gender?
   - ☐ Male
   - ☐ Female
   - ☐ Other __________________

…………………………………………………………………………………………………

4. What is your ethnicity?
   - ☐ Hispanic or Latino/Latina
   - ☐ Non-Hispanic or Latino/Latina

…………………………………………………………………………………………………

5. What is your race?
   - ☐ American Indian or Alaska Native
   - ☐ Asian
   - ☐ Black or African American
   - ☐ Native Hawaiian or Other Pacific Islander
   - ☐ White
   - ☐ Other ___________________

…………………………………………………………………………………………………

6. What is your class?
   - ☐ Freshmen
   - ☐ Sophomore
   - ☐ Junior
   - ☐ Senior
   - ☐ Graduate

…………………………………………………………………………………………………

7. Have you been encouraged by a health care provider to take COVID-19 vaccine?
   - ☐ Yes
   - ☐ No

…………………………………………………………………………………………………

8. What is your political affiliation?
   - ☐ Republican
   - ☐ Democratic
   - ☐ Independent
   - ☐ Other

…………………………………………………………………………………………………

9. What is your religion?
   - ☐ Christianity
   - ☐ Islam
   - ☐ Buddhism

- ☐ Judaism
- ☐ Atheist
- ☐ Hinduism
- ☐ Other

10. Where do you live?
- ☐ On-campus
- ☐ Off-campus

11. What is your current overall GPA? (on a 4.00 scale)
- ☐ Less than 1.99
- ☐ 2.00 – 2.49
- ☐ 2.50 – 2.99
- ☐ 3.00 – 3.49
- ☐ 3.50 – 4.00

12. Do you have health insurance?
- ☐ Yes
- ☐ No

13. Do you work for pay?
- ☐ No
- ☐ Yes, How many hours? __________

14. What is your marital status?
- ☐ Married
- ☐ Divorced
- ☐ Widowed
- ☐ Separated
- ☐ Never married
- ☐ In a civil union or registered domestic partnership
- ☐ A member of an unmarried couple

	**Never**	**Almost Never**	**Sometimes**	**Fairly Often**	**Very Often**
15. Taking the COVID-19 vaccine will protect me against coronavirus infection.	☐	☐	☐	☐	☐
16. Taking the COVID-19 vaccine will protect my family from getting coronavirus infection.	☐	☐	☐	☐	☐
17. Taking the COVID-19 vaccine will allow me to resume my daily activities.	☐	☐	☐	☐	☐

	Never	Almost Never	Sometimes	Fairly Often	Very Often
18. Taking the COVID-19 vaccine may not be safe.	☐	☐	☐	☐	☐
19. It bothers me that long-term studies have not been done on COVID-19 vaccine.	☐	☐	☐	☐	☐
20. It concerns me that COVID-19 vaccine may become ineffective due to mutation of the virus.	☐	☐	☐	☐	☐

	Not At All Sure	Slightly Sure	Moderately Sure	Very Sure	Completely Sure
21. How sure are you that you can take COVID-19 vaccine, if it is available today?	☐	☐	☐	☐	☐
22. How sure are you that you can complete the 2-shot course of COVID-19 vaccine despite getting side effects, if it is available today?	☐	☐	☐	☐	☐
23. How sure are you that you can complete the 2-shot course of COVID-19 vaccine despite worries about long term studies, if it is available today?				☐	☐

	Not At All Sure	Slightly Sure	Moderately Sure	Very Sure	Completely Sure
24. How sure are you that you will have access to the COVID-19 vaccine when it is available to you?	☐	☐	☐	☐	☐
25. How sure are you that you will be able to afford the COVID-19 vaccine when it is available to you?	☐	☐	☐	☐	☐
26. How sure are you that you will be able to go and get the COVID-19 vaccine when it is available to you?	☐	☐	☐	☐	☐

	Not At All Likely	Somewhat Likely	Moderately Likely	Very Likely	Completely Likely
27. How likely is it that you will take COVID-19 vaccine when it is available to you?	☐	☐	☐	☐	☐

Thank you for your time!

SCORING GUIDE

Construct of advantages: Rate items 15-17 on a scale of 0-4 and then sum to derive a possible score of 0-12 units.

Construct of disadvantages: Rate items 18-20 on a scale of 0-4 and then sum to derive a possible score of 0-12 units.

Construct of participatory dialogue: Subtract disadvantages from advantages score to derive a possible score of -12 to + 12 units .

Construct of behavioral confidence: Rate items 21-23 on a scale of 0-4 and then sum to derive a possible score of 0-12 units.

Construct of changes in the physical environment: Rate items 24-26 on a scale of 0-4 and then sum to derive a possible score of 0-12 units.

Construct of initiation intention: Rate item 27 on a scale of 0-4 and derive a possible score of 0-4 units.

Flesch Reading Ease: 63.5
Flesch-Kincaid Grade Level: 5.9

© Manoj Sharma
